# Cecal Perforation by a Large Gallstone: An Unusual Diagnosis

**DOI:** 10.7759/cureus.7859

**Published:** 2020-04-27

**Authors:** Mariana Claro, Daniel C Santos, Diogo Sousa, Manuel Colaço, José Augusto Martins

**Affiliations:** 1 General Surgery, Hospital do Litoral Alentejano, Santiago do Cacém, PRT

**Keywords:** cholecystocolic fistula, colonic perforation, gallstone colonic obstruction, rigler´s triad

## Abstract

Cholecystocolic fistulas are uncommon, with rare cases of colonic obstruction described in the literature and even rarer cases of intestinal perforation due to gallstones.

We describe a case of a 73-year-old man who presented to our ED with complaints of diffuse abdominal pain, vomiting, constipation, and fever for the past week. Abdomen CT showed signs of acute perforated appendicitis. An exploratory laparotomy was proposed which revealed cecal perforation caused by a 3 cm gallstone. A right colectomy was performed with primary anastomosis, without cholecystectomy or fistula repair. The postoperative period was complicated due to an anastomotic dehiscence on day 12 with the need for a re-laparotomy with an ileotransverse colostomy confection. The patient was in the ICU care for five days and was discharged on the 13th day after the second intervention.

The clinical presentation of gallstone ileus is nonspecific and vague often leading to a delay in the diagnosis and treatment. CT scan has the best specificity and sensibility for the diagnosis but abdominal X-ray may show the pathognomonic Rigler´s triad. The surgical treatment consists of removing the gallstone with or without simultaneous cholecystectomy and fistula repair.

Reports of colonic perforation due to gallstones are very scarce, which makes this a very low suspicion diagnosis. The ideal surgical approach is not established. The morbidity of these cases can reach 50%.

## Introduction

Gallstone disease is a common condition known to predominantly affect female patients [1]. It can occur in a wide spectrum of forms, ranging from asymptomatic to biliary colic, biliary tree obstruction with or without cholangitis and cases of acute or chronic cholecystitis. Gallstone ileus is a rare complication of gallstone disease, developing in 0.3%-0.5% of patients [2], with a higher frequency among the elderly [3]. It is defined as a mechanical bowel obstruction caused by the impaction of a large gallstone that has passed through a fistula between the gallbladder and the gastrointestinal tract. Bowel perforation can be associated in very few cases.

Signs and symptoms of gallstone ileus are nonspecific, with reports of nausea, vomiting and abdominal pain, often leading to a delay in the diagnosis and prompt treatment. More than 50% of cases are discovered only during laparotomy [1]. The lack of case reports and studies in the literature contributes to the uncertainty around the best emergency surgical treatment of gallstone ileus. The resolution of the gastrointestinal obstruction is the mainstay of treatment, whereas the need for definitive repair of the biliary fistula remains an open debate.

## Case presentation

**Figure 1:**
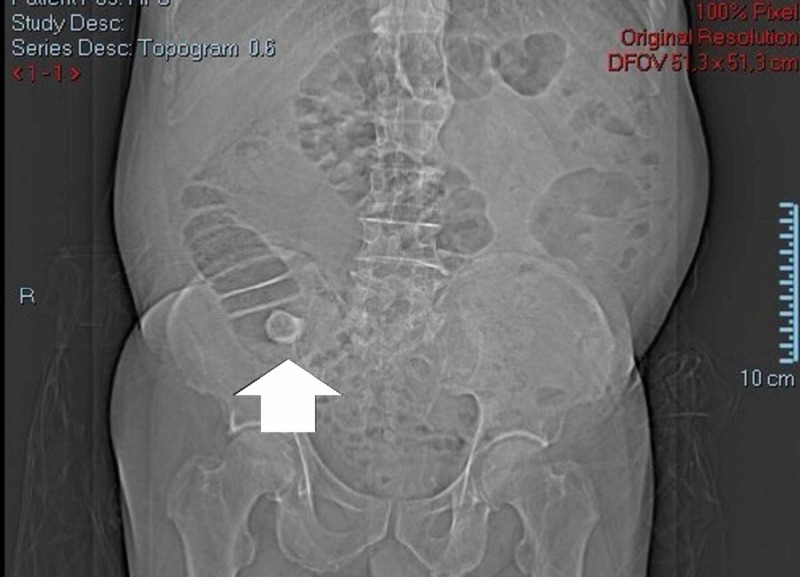
CT scan view. One of Rigler´s triad signs - presence of ectopic gallstone.

**Figure 2:**
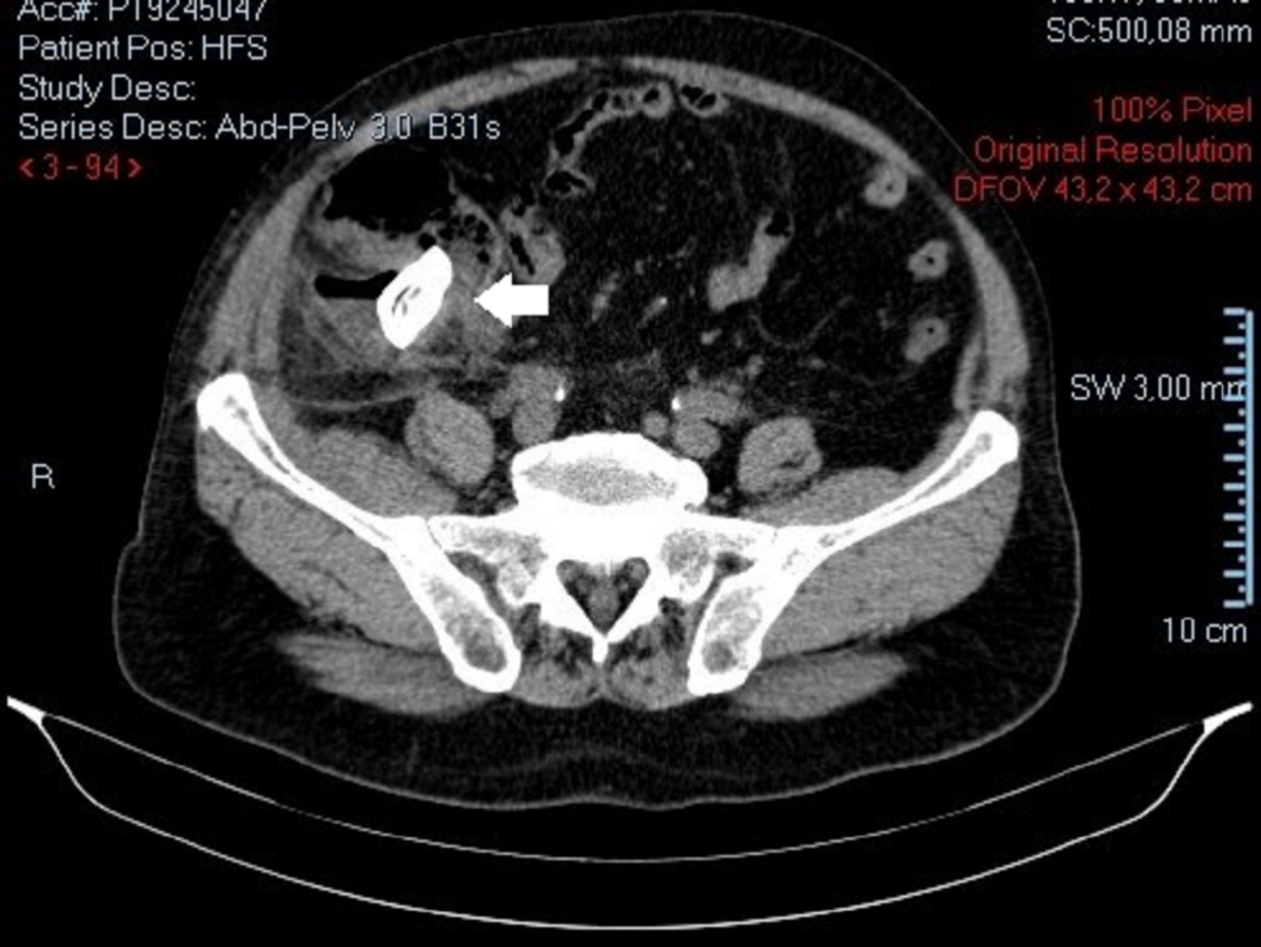
Abdominopelvic CT scan. Presence of a presumed "appendicolith" of 35 mm at the cecum near the base of the ileocecal appendix.

**Figure 3:**
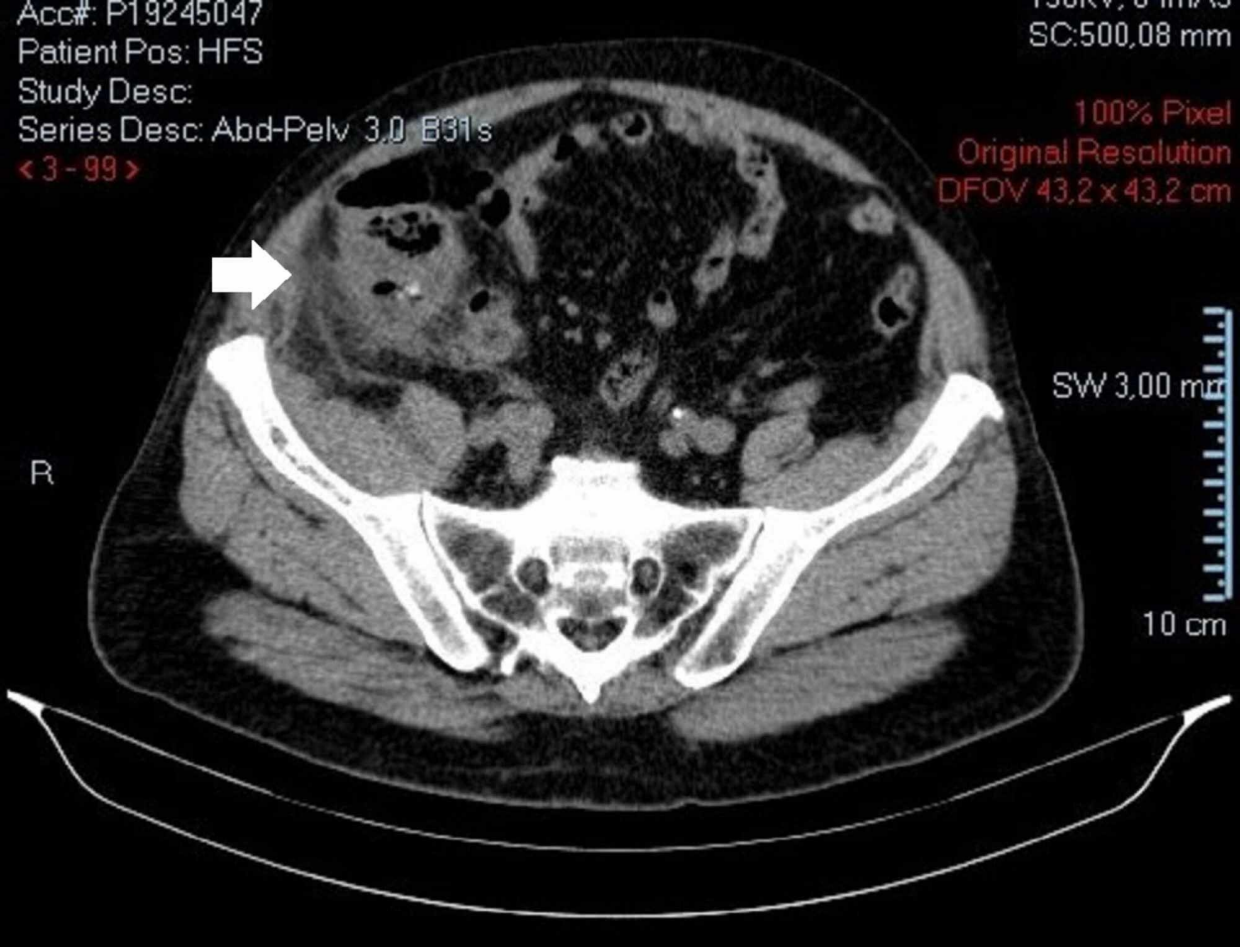
Abdominopelvic CT scan. Fat stranding and parietal peritoneal thickening in parietocolic gutter.

A 73-year-old male with a personal history of ischemic heart disease, chronic pulmonary obstructive disease, hypertension and a previous partial gastrectomy with a Billroth II reconstruction due to a peptic ulcer, presented to our ED with a one-week history of diffuse abdominal pain, vomiting, constipation, and fever. Physical examination revealed a distended, tender abdomen, with pain upon palpation of the right lower quadrant, showing clinical signs of peritonitis. Laboratory data showed a slightly elevated white blood cell count of 11.9 x 103/μL, hemoglobin of 13.1g/dL, platelet count of 269 x 103/μL, normal renal function with urea 48 mg/dL, creatinine 1.3 mg/dL, and a C-reactive protein of 28.60 mg/dL (normal range <0.5) without electrolyte or liver enzymes alterations. The patient performed a CT scan (Figures 1-3) which revealed a distended ileocecal appendix on the right iliac fossa (25 mm), with an appendicolith of 35 mm on its base, and a collection of 26 mm x 20 mm with an air-fluid level suggestive of an acute appendicitis complicated by rupture and abscess formation.

**Figure 4:**
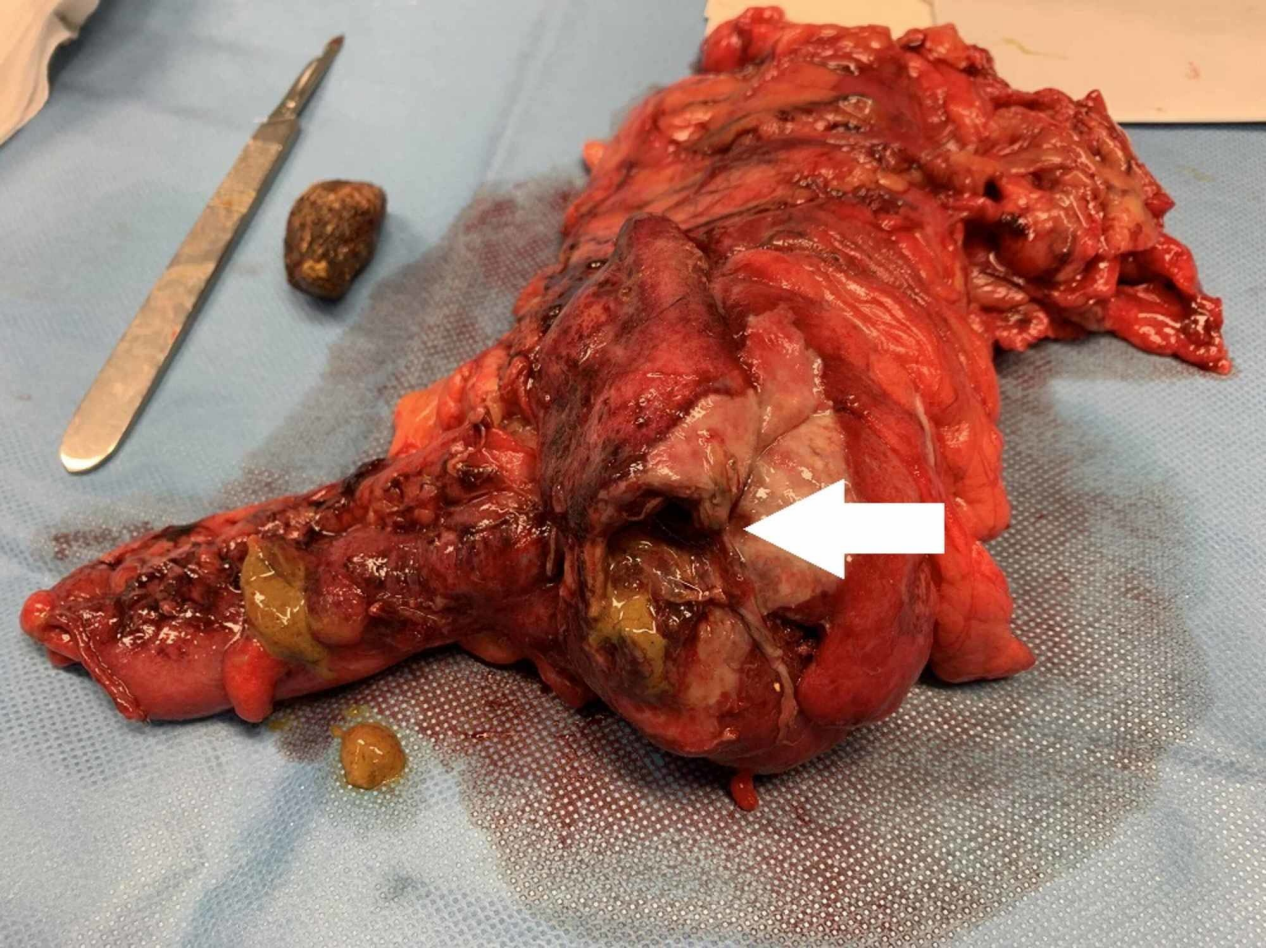
Right hemicolectomy with cecal perforation.

**Figure 5:**
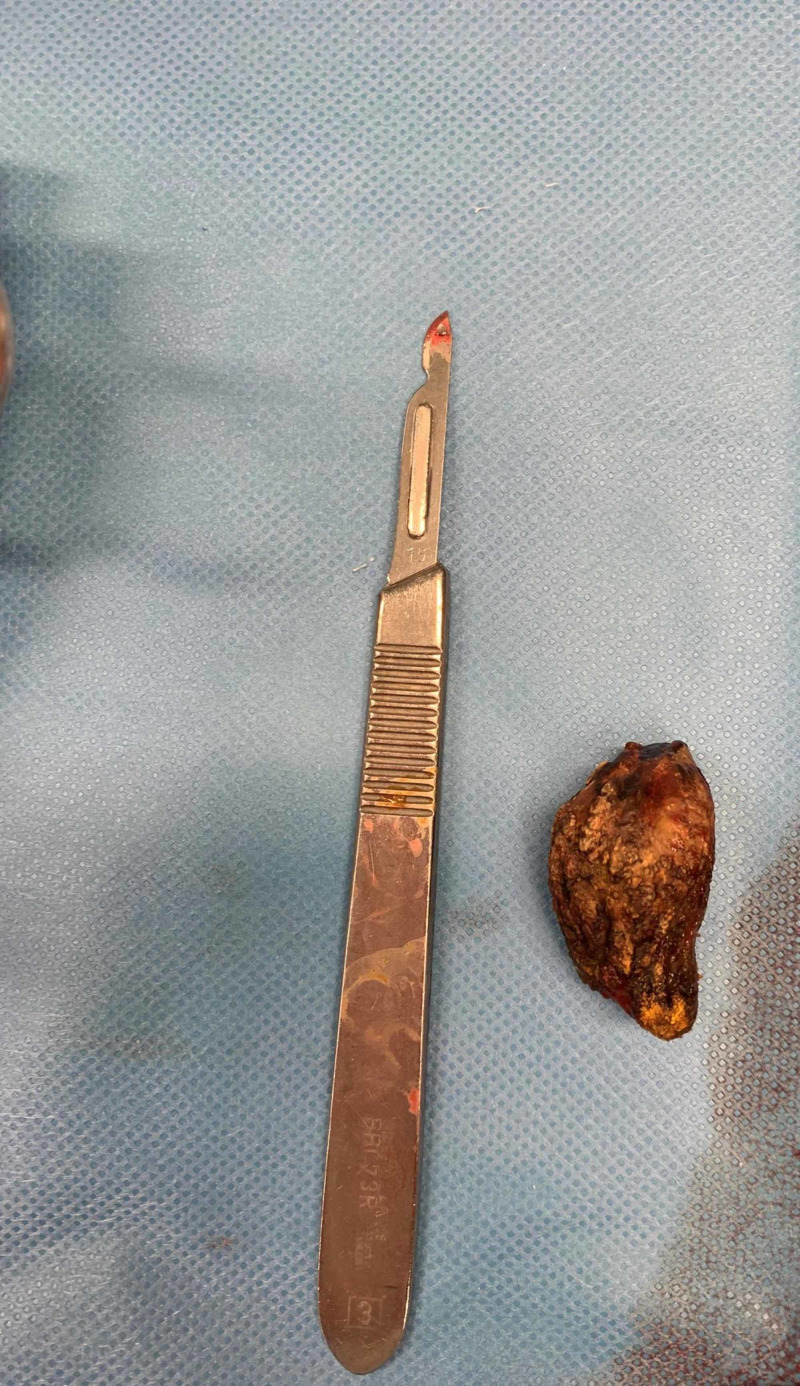
Gallstone retrieved from the abdominal cavity with approximately 30 mm.

An exploratory laparotomy was performed evidencing cecal perforation caused by a biliary stone with approximately 30 mm with a contiguous abscess along with multiple adhesions between the transverse colon and the gallbladder which complicated the positive identification of the cholecystocolonic fistulous tract. The decision was to perform a right hemicolectomy with primary anastomosis without cholecystectomy or fistula repair, and placement of a suction drain on the right paracolic gutter (Figures 4-5).

**Figure 6:**
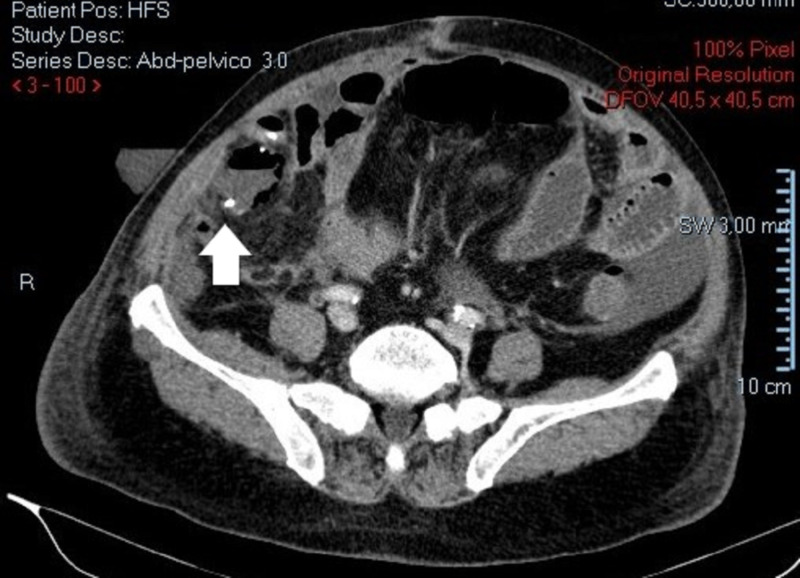
Contrast enhanced abdominopelvic CT scan. Free air around previous site of colonic anastomosis consistent with anastomotic dehiscence.

**Figure 7:**
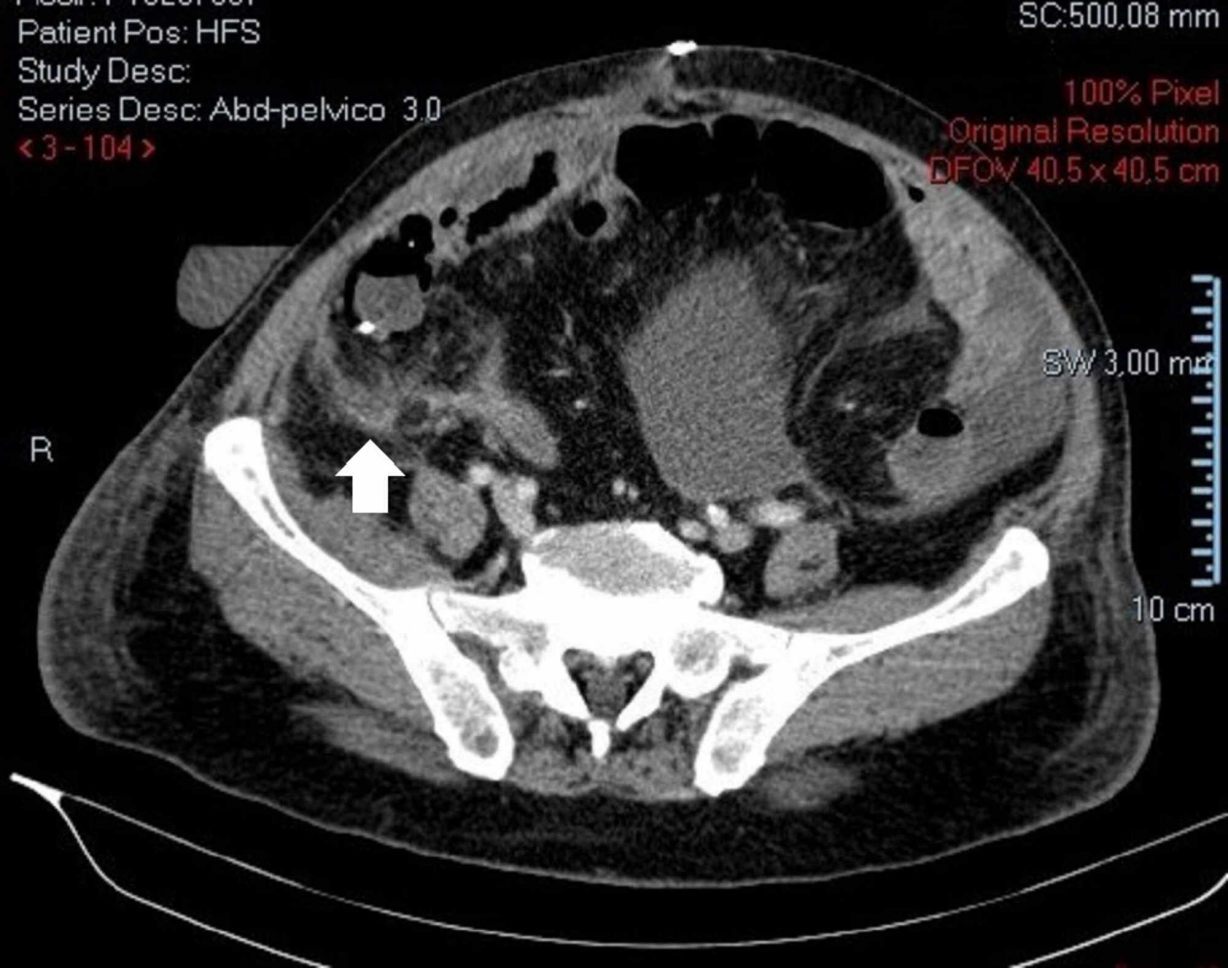
Contrast enhanced abdominopelvic CT scan. Peritoneal thickening with contrast uptake and mesenteric fat densification suggestive of peritonitis.

**Figure 8:**
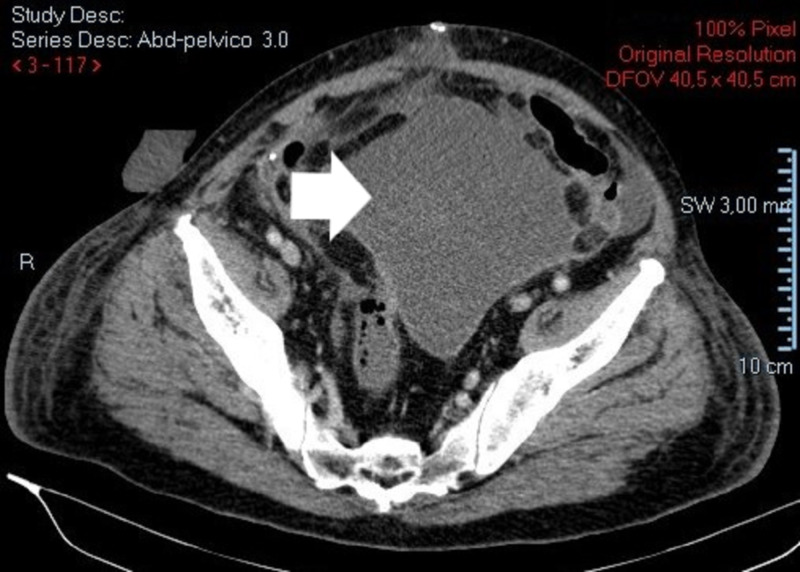
Contrast enhanced abdominopelvic CT scan. Intraperitoneal loculated free fluid.

The patient was started on intravenous antibiotics and resumed bowel function on the eighth postoperative day. However, by that time, he started to experience fever with increased inflammatory markers. On the 12th postoperative day, biliary fluid drainage was noticed. A CT scan with intravenous contrast was performed which suggested an anastomotic dehiscence (Figures 6-8).

The patient was consequently taken to an emergency re-laparotomy, which confirmed the diagnosis. A resection of the previous anastomosis and the creation of an ileotransverse colostomy were performed. The patient was admitted to the ICU in the immediate postoperative period, with rapid progression to septic shock. He responded to aggressive resuscitation measures and was transferred to the surgical ward on the fifth day after the intervention. The rest of the postoperative period was uneventful and the patient was discharged on the 13th day (post last intervention). The patient has not had more complaints since the discharge and is being followed at our outpatient clinic for programming re-establishment of intestinal continuity.

## Discussion

The presentation of gallstone ileus may be preceded by a history of biliary symptoms, which can be absent in up to one third of the cases, with intestinal obstruction with vomiting and abdominal pain being the most common clinical presentation.

Laboratory tests may reveal slight leukocytosis, electrolyte imbalance, signs of dehydration and abnormalities in liver function in up to 41% of patients, which are of little diagnostic value [4]. 

In 1941 Rigler described a triad composed by three pathognomonic radiological signs: 1) aerobilia, 2) bowel obstruction, and 3) presence of an ectopic gallstone. However, these features are only present in one third of patients [5]. Contrast enhanced CT is considered superior to plain abdominal films or ultrasound (US) in the diagnosis of gallstone ileus, with a sensitivity, specificity, and accuracy of up to 93%, 100% and 99%, respectively [6], also allowing for detection of edema and possible bowel ischemia.

Cholecystoenteric fistulas are a rare entity, with an incidence of 0.4%-1.9% [7]. Fistulation is presumably related to an inflammatory reaction, caused by either an acute episode of cholecystitis or a more indolent chronic subclinical inflammation of the gallbladder. The inflammation and the pressure exerted by the gallstones are responsible for the erosion of the gallbladder wall, creating a fistula between this organ and the gastrointestinal tract, thus promoting the passage of the stones. Cholecystoduodenal fistulas are the most common type, due to their proximity, with cholecystocolonic fistulas accounting for only 8%-26.5% of all cholecystoenteric fistulas [7].

Gallstones larger than 2 cm [4] may impact in any part of the gastrointestinal tract, with terminal ileum and the ileocecal valve being the most common places (80.1%) due to their narrowed lumens and reduced peristalsis [8]. On the other hand, colonic obstruction is only seen in around 4.1% of cases [8]. The pressure made by the gallstone on the bowel wall may develop into ischemia, with subsequent necrosis and bowel perforation.

Gallstone ileus causing obstruction of the colon is a rare condition with cases of colonic perforation due to gallstones being even rarer. A literature research showed only six reported cases of colonic obstruction and perforation attributable to gallstones, all involving the sigmoid colon [7-12]. Hence, to the best of our knowledge, this is the first reported case concerning cecal perforation by gallstones. Another remarkable feature about this case was that our patient never experienced a typical bowel obstruction and gallstone ileus presentation. We believe that the pressure exerted by the stone on the base of the caecum or the base of the ileocecal appendix was the cause of the bowel rupture, leading to peritonitis rather than a mechanical intestinal obstruction. 

Gallstone ileus is a condition most commonly seen in the population over 65 years [1], usually with other co-morbid conditions, which can have an impact on treatment options.

Less invasive techniques such as use of laxatives, colonoscopy retrieval of the impacted stone and gallstone fragmentation using lithotripsy have been successfully employed in some selected cases, depending on the gallstone’s sizes and composition [13].

Surgery remains the mainstay of treatment although, due to the lack of case reports and case studies, no formal recommendations exist on optimal management in an acute setting. The current surgical options are: 1) enterolithotomy, 2) enterolithotomy, cholecystectomy, and fistula repair (one-stage procedure) and 3) enterolithotomy with a deferred cholecystectomy and fistula repair after four to six weeks (second-stage procedure) [1, 14]. Bowel resection may be required in cases of perforation or necrosis, with either end-to-end anastomosis or a colostomy confection, depending on the grade of fecal contamination of the peritoneal cavity [10].

A retrospective study conducted by Tan et al. studied 19 consecutive patients treated in an emergency setting for gallstone ileus, comparing enterolithotomy alone and enterolithotomy combined with definitive biliary tract surgery and fistula closure. They concluded that the one-stage procedure, despite associated with prolonged operating time, should be offered to patients with lesser comorbidities and a better ASA score, thus excluding the risks related to the persistence of a bilioenteric fistula such as recurrent gallstone ileus, cholecystitis, and recurrent cholangitis [14].

In contrast, on the largest review of published reports on gallstone ileus, Reisner and Cohen reported a lower morbidity after enterolithotomy alone, with a <5% risk of gallstone ileus recurrence and only 10% of patients requiring surgical re-intervention for persistent symptoms. They also reported a lower mortality rate associated with the enterolithotomy group (11.7%), compared to 16.7% of those who underwent the one-stage procedure [1]. Therefore, considering the majority of patients presenting with gallstone ileus are elderly with considerable co-morbidities and that the diagnosis is often delayed leading to dehydration, shock, sepsis or peritonitis [2], relief of gastrointestinal obstruction by simple enterolithotomy should be considered as the safest surgical option in these cases.

Halabi et al. also determined that when bowel resection was needed, it was associated with higher mortality rates than enterolithotomy alone [15].

The morbidity described in these patients can reach 50%, with wound infection being reported as the most common postoperative complication [16], despite the use of systemic antibiotic treatment [4]. Martínez Ramos et al. found a 100% complication rate in patients requiring intestinal resection [17].

All of the previous reported cases found in the literature involving sigmoid perforation by gallstones [7-12] referred to elderly patients with multiple co-morbidities and, in all cases, decision was made to perform a Hartmann´s procedure. However, in only one of the reported cases [11], a second-stage procedure was performed, with cholecystectomy and closure of the cholecystocolonic fistula made at the same time as restoration of the intestinal continuity.

In this case, we chose to only address the cecal perforation with a right colectomy considering our patient´s age and previous co-morbidities and the fact that given the multiple adhesions found on the supramesocolic compartment, trying to correct the cholecystocolic fistula could add significant morbidity to the procedure. 

In light of the current evidences, relief of the intestinal obstruction remains the goal of the treatment as, in the long term, most of the remnant fistulas appear to close spontaneously, particularly when the cystic duct is patent and there are no residual gallstones [4, 14], not leading to further complications. Delayed fistula-oriented interventions may be considered when the risk of recurrence overweighs the risk of surgery [18].

## Conclusions

Gallstone ileus is a rare clinical manifestation that must be taken into consideration when approaching elderly patients with symptoms consistent with bowel obstruction and recurrent cholecystitis. Contrast enhanced CT scan is the best diagnostic test to be used. Treatment plan must be tailored to each patient according to age and comorbidities, with the management of bowel obstruction remaining the cornerstone of treatment. In cases where bowel resection is indicated, there is a higher morbidity and mortality involved.
